# Discovery of novel plastid phenylalanine (*trn*F) pseudogenes defines a distinctive clade in Solanaceae

**DOI:** 10.1186/2193-1801-2-459

**Published:** 2013-09-12

**Authors:** Péter Poczai, Jaakko Hyvönen

**Affiliations:** Plant Biology, Department of Biosciences, University of Helsinki, PO Box 65, Helsinki, FIN 00014 Finland

**Keywords:** Chloroplast DNA (cpDNA), Gene duplications, Phylogeny, Plastome evolution, Tandem repeats, *trn*L-*trn*F, Solanaceae

## Abstract

**Background:**

The plastome of embryophytes is known for its high degree of conservation in size, structure, gene content and linear order of genes. The duplication of entire tRNA genes or their arrangement in a tandem array composed by multiple pseudogene copies is extremely rare in the plastome. Pseudogene repeats of the *trn*F gene have rarely been described from the chloroplast genome of angiosperms.

**Findings:**

We report the discovery of duplicated copies of the original phenylalanine (*trn*F_GAA_) gene in Solanaceae that are specific to a larger clade within the Solanoideae subfamily. The pseudogene copies are composed of several highly structured motifs that are partial residues or entire parts of the anticodon, T- and D-domains of the original *trn*F gene.

**Conclusions:**

The Pseudosolanoid clade consists of 29 genera and includes many economically important plants such as potato, tomato, eggplant and pepper.

**Electronic supplementary material:**

The online version of this article (doi:10.1186/2193-1801-2-459) contains supplementary material, which is available to authorized users.

## Findings

The plastid *trn*T-*trn*F region has been widely applied to resolve phylogeny of embryophytes (Quandt and Stech 2004; Zhao et al. 2011) and to address various questions of population genetics since the development of universal primers by Taberlet et al. (1991). This marker is located in the large single copy region of the chloroplast genome and contains a co-transcribed region consisting of three highly conserved exons that code the transfer RNA (tRNA) genes for threonine (UGU), leucine (UAA) and phenylalanine (GAA). The region is interspersed by two intergenic spacers and by a group I intron intercalated within the first and second exon of the *trn*L_(UAA)_ gene. Phylogenetic results obtained with the *trn*T-*trn*F region (or part of it) should be treated with caution. This is due to the fact that some recent studies (e.g. Koch et al. 2005; Pirie et al. 2007; Schmikl et al. 2009; Vivjerberg and Bachmann 1999) have shown that there are clearly several copies of certain parts of this region. If this is ignored, it will easily lead to situations where basic requirement of homology of the characters used for phylogenetic analyses is compromised. This might lead to false hypotheses of phylogeny, especially when they are based on the analyses of only this region.

Larger structural changes (>50 bp) rarely occur in the plastome. However, duplications of the *rpl*2 or *rpl*23 genes (Bowman et al. 1988) or even the duplication of tRNAs (pseudogenes) are occasionally reported. The later are extremely rare in angiosperms and so far they have only been described from Asteraceae (Vijverberg and Bachmann 1999; Witzell 1999), Annonaceae (Pirie et al. 2007), Brassicaceae (Ansell et al. 2007; Koch et al. 2007; Tedder et al. 2010) and Juncaceae (Drábkova et al. 2004). In our recent study we reported a tandem repeat comprising of two to four pseudogene copies upstream of the original *trn*F gene in four *Solanum* (Solanaceae) species (Poczai and Hyvönen 2011a). We have characterized these structural duplications and shown that they consist of several highly structured motifs, which are partial residues, or entire parts of the anticodon, T- and D-domains of the original gene, but all lack the acceptor stems at the 5′ or 3′. We were further interested to evaluate the possible occurrence of complete or partial *trn*F pseudogenes in Solanaceae. This family contains many economically important plant species, e.g., potato (*Solanum tuberosum* L.), tomato (*Solanum lycopersicum* L.) and paprika (*Capsicum annuum* L.) and is under intensive phylogenetic investigation and the *trn*T-F plastid marker is commonly used in these studies. These sequences together with the results of molecular breeding programs provide large amount of data that is available in GenBank. During data mining we concentrated on a structured dataset generated in previous phylogenetic studies (Fukuda et al. 2001; Garcia and Olmstead 2003; Santiago-Valentin and Olmstead 2003; Bohns 2004; Clarkson et al. 2004; Levin and Miller 2005; Levin et al. 2005; Weese and Bohns 2007; Olmstead et al. 2008) that contained 195 taxa and 390 sequences. This dataset provided the basis for the latest robust phylogenetic hypothesis of the Solanaceae including 89 from the 98 (Olmstead and Bohns 2007) recognized genera. Manual search using the anticodon domain of the original *trn*F gene and automated tRNA recognition by CENSOR (Kohany et al. 2006) indicated the presence of pseudogene repeats in numerous genera of Solanaceae.

**Figure 1:**
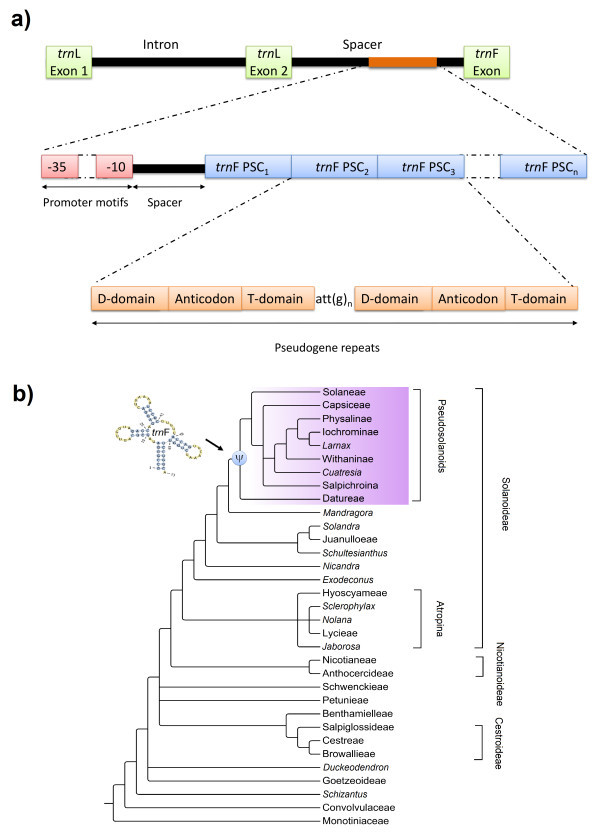
**Phylogeny of Solanaceae and the distribution and schematic structure of*trn*F pseudogene copies. a)** Suprageneric groups recognized are indicted to the right on the tree, while major clades are collapsed at the base node and their names follow Olmstead et al. (2008). The new Pseudosolanoid clade united by the presence of pseudogenic *trn*F gene duplication is marked with ‘ψ’ in the Solanoideae subfamily. **b)** The schematic representation of the plastidic *trn*L-F spacer region in Solanaceae and the intercalated pseudogene copies (PSC) in the intergenic spacer region close to 5′ of the *trn*F gene. Pseudogene repeats are variable in number and structure and are found after the putative promoter motifs that are also variable among species. The spacer region between the first PSC and promoter motifs consists of intergenic repeats of variable length. Each PSC is separated by a common bordering motif (ATTG) at the 5′end.

We used the core *trn*L-F dataset to map the occurrence of pseudogenic repeats on the phylogenetic tree of Solanaceae. As presented in Figure 1 the distribution of pseudogenic duplications is in congruence with the previously published phylogeny of the Solanaceae (Olmstead et al. 2008), and it is obvious that the first pseudogenic copy evolved only once at the base of a highly supported clade within the subfamily Solanoideae. Among the members of this lineage, referred here as the Pseudosolanoid clade, the anticodon domain of the *trn*F gene exhibits extensive gene duplications with one to seven tandemly repeated copies in close 5′-proximity of the original functional gene (Table 1). The size of each pseudogenic copy ranged between 32 and 73 bp and the anticodon domain was identified as the most conserved element. A common ATT(G)_n_ motif is of particular interest and its modifications were found to border the 5′ of the duplicated regions in the same way as found in Brassicaceae (Ansell et al. 2007; Koch et al. 2005 and 2007; Schmikl et al. 2009; Tedder et al. 2010). Other motifs were partial residues or entire parts of the T- and D-domains. The residues of the 3′ and 5′ acceptor stems were rarely found among the copies (see Table 1). The D-domain was more conserved than the T-domain among the copies and other internal repeats (AT, AAT, ATT, AATCC) were intercalated within this region for example in genus *Lycianthes* (Dunal.) Hassl. In addition to these newly discovered pseudogenes we were also able to characterize putative promoter motifs showing high similarity to a sigma^70^-type bacterial promoter. These two elements (−35 TTGACA/-10 GAGGAT) are consistently found in the *trn*L-F spacer region of embryophytes, and they are believed to represent the ancient and original *trn*F gene promoter (Quandt et al. 2004). Interestingly, pseudogenic repeats were found to be exclusively inserted after such motifs in Solanaceae, contrary to Brassicaceae, where similar pseudogenic repeats were found only between promoter motifs in the *trn*L-F intergenic spacer region (Koch et al. 2005). The later finding lead Koch et al. (2005) to support the conclusion by Kanno and Hirtai (1993) that these elements should be non-functional due to the intercalated position of pseudogenes between promoters. However, this may be challenged by the position of Solanaceae pseudogenes following the −10 and −35 promoters, which are also variable in number and composition.

**Table 1:** **Distribution of*trn*F pseudogenes among Solanaceae and number of multiplicated*trn*F anticodon domains**

Taxa	GenBank	Tribe	Copy number
*Acnistus arborescens*	EU580954	Physaleae	2^a,b^
*Aureliana fasciculata*	EU580961	Physaleae	2
*Brachistus stramonifolius*	EU580963	Physaleae	3
*Brugmansia aurea*	EU580965	Datureae	1^c^
*Brugmansia sanguinea*	EU580966	Datureae	1^c^
*Capsicum baccatum*	EU580969	Capsiceae	4^a,b^
*Capsicum chinense*	EU603443	Capsiceae	4^d^
*Capsicum minutiflorum*	EU580970	Capsiceae	6^d^
*Capsicum pubescens*	AY348982	Capsiceae	6^b,d^
*Capsicum rhomboideum*	EU580971	Capsiceae	1^e^
*Chamaesaracha coronopus*	EU580978	Physaleae	4
*Chamaesaracha sordida*	EU580979	Physaleae	4
*Cuatresia exiguiflora*	EU580981	Physaleae	2
*Cuatresia riparia*	EU580982	Physaleae	2
*Datura leichhardtii*	EU580983	Datureae	1^f^
*Datura stramonium*	EU580984	Datureae	1^f^
*Deprea sylvarum*	EU580985	Physaleae	3
*Discopodium penninervum*	EU580986	Physaleae	4
*Dunalia solanacea*	EU580988	Physaleae	4
*Eriolarynx lorenzii*	EU580990	Physaleae	4
*Iochroma australe*	EU580999	Physaleae	4
*Iochroma cardenasianum*	EU581000	Datureae	1^f^
*Iochroma fuchsioides*	EU581001	Physaleae	2
*Iochroma umbellatum*	EU581002	Physaleae	2
*Jaltomata auriculata*	EU581006	Solaneae	2^f^
*Jaltomata grandiflora*	EU581007	Solaneae	2^f^
*Jalotmata procumbens*	AY098695	Solaneae	1^a,b,f^
*Jaltomata sinuosa*	DQ180418	Solaneae	2^f^
*Larnax subtriflora*	EU581009	Physaleae	3
*Leucophysalis grandiflora*	EU581013	Physaleae	2
*Leucophysalis nana*	EU581014	Physaleae	2
*Lycianthes biflora*	EU581015	Capsiceae	2^g^
*Lycianthes ciliolata*	EU581016	Capsiceae	4^h,i^
*Lycianthes glandulosa*	EU581017	Capsiceae	3^g,i^
*Lycianthes heteroclita*	DQ180414	Capsiceae	2^g,i^
*Lycianthes inaequilatera*	EU581018	Capsiceae	6^h^^,^^i^
*Lycianthes multiflora*	EU581019	Capsiceae	3^g^^,^^i^
*Lycianthes peduncularis*	EU581020	Capsiceae	4^h,i^
*Lycianthes shanesii*	EU581021	Capsiceae	1^g,h,i^
*Margaranthus solanaceus*	EU581025	Physaleae	5^i^
*Nectouxia formosa*	EU581031	Salpichroina*	1^a,b^
*Nothocestrum latifolium*	EU581037	Physaleae	2
*Nothocestrum longifolium*	EU581038	Physaleae	3
*Oryctes nevadensis*	EU581039	Physaleae	3
*Physalis alkekengi*	DQ180420	Physaleae	2
*Physalis carpenteri*	EU581042	Physaleae	2
*Physalis heterophylla*	EU581043	Physaleae	2
*Physalis peruviana*	EU581044	Physaleae	4^a,b^
*Physalis philadelphica*	EU581045	Physaleae	5^a,b^
*Quincula lobata*	EU581051	Physaleae	1^a,b^
*Salpichroa origanifolia*	EU581052	Salpichroina*	2^a,b^
*Saracha punctata*	EU581053	Physaleae	4
*Solanum abutiloides*	AY266236	Solaneae	1^f^
*Solanum aviculare*	HM006836	Solaneae	2^a,b,f^
*Solanum betaceum*	DQ180426	Solaneae	1^f^
*Solanum dulcamara*	HM006840	Solaneae	1^f^
*Solanum herculeum*	DQ180466	Solaneae	2^f^
*Solanum lycopersicum*	NC007898	Solaneae	1^f^
*Solanum melongena*	EU176149	Solaneae	2^h,i^
*Solanum pseudocapsicum*	DQ180436	Solaneae	1
*Solanum torvum*	AY266246	Solaneae	4^i^
*Solanum trisectum*	JN130370	Solaneae	2
*Solanum wendlandii*	DQ180440	Solaneae	1
*Tubocapsicum anomalum*	EU581066	Physaleae	7
*Vassobia dichotoma*	EU581067	Physaleae	4
*Witheringia cuneata*	EU581070	Physaleae	2
*Witheringia macrantha*	EU581071	Physaleae	5
*Witheringia meiantha*	EU581072	Physaleae	4
*Witheringia mexicana*	EU581073	Physaleae	5
*Witheringia solanacea*	EU581074	Physaleae	3

Taxonomic classification and a GenBank accession number is provided for each species. *Unranked informal clade name.

^a^Partial pseudogenic copy at the 3′ end. ^b^Missing original *trn*F gene. ^c^Intact 5′acceptor stem present. ^d^Three copies of −35 promoter (TTGACA) motif. ^e^Four copies of −35 promoter (TTGACA) motif. ^f^One copy of −10 promoter (GAGGAT) motif present. ^g^Pseudogene repeats are separated by long internal repeats after the promoter motifs. ^h^Only one copy of −35 promoter (TTGACA) motif. ^i^3′ acceptor stem present.

The occurrence of pseudogenes provides strong evidence of relationships among some groups that had low support values in the previous analyses (e.g. Olsmtead et al. 2008). This event robustly separates the *(1)* Atropina (Hyoscyameae, Lycieae, Jabrosa, Latua, Nolana and Scleraphylax) and *(2)* Juanulloeae clades from the Pseudosolanoid clade composed by *(3)* Solaneae, Capsiceae, Physaleae and Datureae and *(4)* Salpichroina (*Salpichroa* Miers and *Nectouxia* Kunth). In clades *(1)* and *(2)* pseudogenes are absent while they appear at the basal node of clade *(3)* and *(4).* This lineage where pseudogene copies have been found includes 29 genera; here belongs also the clade of *Solanum* L. and *Capsicum* L. with many economically important plant species. However, sequence information was lacking for the genera *Mellissia* Hook. f. and *Athenaea* Adans. to confirm the presence of *trn*F pseudogenes. This is not surprising as available plant material of these taxa is very restricted. For example *Mellissia* is a genus with a single species, *Mellissia begoniifolia* (Roxb.) Hook. f. which is critically endangered and endemic to the island of Saint Helena. The larger clade of Solanoideae also includes several branches with low support values composed of small genera (*Exodeconus* Raf., *Mandragora* L., *Nicandra* (L.) Gaerten., *Schultesianthus* Hunz., *Solandra* Sw.) in the phylogeny proposed by Olmstead et al. (2008). These lineages are from the early diversification of the Solanoideae with no close relatives and all lack pseudogene repeats that could be informative to trace their ancestry.

The latest large scale phylogenetic analysis of the Solanaceae (Olmstead et al. 2008) established major clades of the family but sampling in some of the lineages can still be improved. Goldberg et al. (2010) analyzed a larger data set but they did not focus on taxonomic relationships but rather on the evolution of self-compatibility. Some studies have attempted to calibrate a molecular clock for various groups within Solanaceae, but all of these used the same (Paape et al. 2008; Poczai and Hyvönen 2011b), or only few fossil records (Dillon et al. 2009; Tu et al. 2010). Fossil record of the Solanaceae has not been reviewed recently. This urges for the re-assessment of the specimens and could potentially provide more robust calibration points for the family (Särkinen, personal communication). Latest current estimates show the age of the Pseudosolanoids to be approximately 20 My (Särkinen, personal communication), and thus the origin of the pseudogene duplications of Solanaceae to be approximately of the same Miocene age as in Brassicaceae (16–21 My; Koch et al. 2005).

## Conclusions

Despite of the extensive studies based on sequence level characters the taxonomy of the Solanaceae is not yet completely understood. However, there is ongoing work on different levels by multiple groups to resolve phylogenetic relationships (Fukuda et al. 2001; Garcia and Olmstead 2003; Santiago-Valentin and Olmstead 2003; Bohs 2004; Clarkson et al. 2004; Levin and Miller 2005; Levin et al. 2005; Weese and Bohs 2007; Olmstead et al. 2008). There are a number of questions that should be answered regarding the discovery of *trn*F pseudogenes, for example: How did the duplications originate? Are the pseudogene copy numbers a useful character for phylogenetic inference? To what extent does the number of pseudogene copies vary within a single species? The evolution and structure of pseudogenic copies should be compared with others reported from different plant families especially from Brassicaceae. The potential of *trn*F pseudogenes as phylogenetic markers need to be investigated further in the future for better understanding of the evolution of Solanaceae. These investigations could answer what are the wider implications of the pseudogene repeats for Solanaceae studies that utilize the *trn*L-F spacer region.

## Methods

### Solanaceae sequence dataset

For the Solanaceae and several outgroups we used the *trn*L-F spacer data assembled by Olmstead et al. (2008). This dataset contained 195 taxa and 390 sequences generated in previous phylogenetic studies (Fukuda et al. 2001; Garcia and Olmstead 2003; Santiago-Valentin and Olmstead 2003; Bohs 2004; Clarkson et al. 2004; Levin and Miller 2005; Levin et al. 2005; Weese and Bohs 2007; Olmstead et al. 2008) and this was used to align and mask pseudogenic copies. The goal was to map the taxonomic distribution of pseudogenes at family level sampling as many genera as possible. This dataset and representative trees used in our study were previously deposited in TreeBASE (ID S2191). This alignment was also used to demonstrate copy number distribution corresponding to the published phylogenetic hypothesis that was not only based on the *trn*L-F spacer information but relied on sequence data from the *ndh*F region.

### Recognition and copy number assessment of the *trn*F_(GAA)_ pseudogenes

The complete chloroplast genome of *Solanum bulbocastanum* Dunal (DQ347958) was used to select the corresponding loci of the *trn*L-*trn*F spacer region (bp positions 48,854 to 49,382), to annotate ambiguous sequences regions, and to ensure that our interpretations are based on homologous positions. Putative pseudogene repeats were identified with screening using Repbase (Jurka 2000) with the “mask pseudogenes” and “report simple repeats” options of the online tool CENSOR (Kobany et al. 2006). This was done to identify repetitive elements by comparing our sequences to known eukaryotic repeats and prototypic sequences stored in Repbase utilizing WU-BLAST. A second search was conducted with FastPCR (Kalendar et al. 2009) using the repeat search option of the program. Under “type of repeats” we checked for simple, direct, inverted, direct antisense, and direct reverse repeats, respectively. Default values were used under a kMers repeat screening. After each search, repetitive motifs and sequences were recorded and compared with the results obtained from the Repbase search. After repeats were identified in the *trn*L-F IGS sequences, further structural *trn*F_(GAA)_ gene elements or residues were annotated manually using the anticodon domain as reference. The annotated sequence alignment is shown in Additional file 1.

### Sequence annotation and alignment

Masked pseudogenic copies were further edited using Geneious v.4.8.5 (Biomatters Ltd.). We used the *Nicotiana tabacum* L. complete chloroplast genome (NC001879; bp positions 49,840 to 50,318) for comparisons and to determine the subunits of pseudogenic repeats as this species lacks these gene duplications. Sequence break points were examined manually to determine the cut off points of pseudogenic copies and to identify bordering motifs. Identified copies were aligned with MUSCLE (Edgar 2004) as implemented in Geneious v.4.8.5 using default settings. The sequence alignment in FASTA format is available as Additional file 2.

## Electronic supplementary material

Additional file 1: **Annotated sequence alignment of pseudogene repeats found in Solanaceae.** Major parts of the *trn*F gene are marked as D- and T-domains and anticodon in the middle together with bordering 5′ and 3′ acceptor stems. The *trn*F gene of *Nicotiana tabacum* is used as a reference sequence to align different pseudogenes. (PDF 4 MB)

Additional file 2: **Sequence alignment of pseudogene copies.** (FASTA 41 KB)
