# Supplementary material for: Discovery of novel plastid phenylalanine (trnF) pseudogenes defines a distinctive clade in Solanaceae
Source: Springerplus. 2013 Sep 12;2:459. doi: 10.1186/2193-1801-2-459 (PMC3786074; doi:10.1186/2193-1801-2-459)

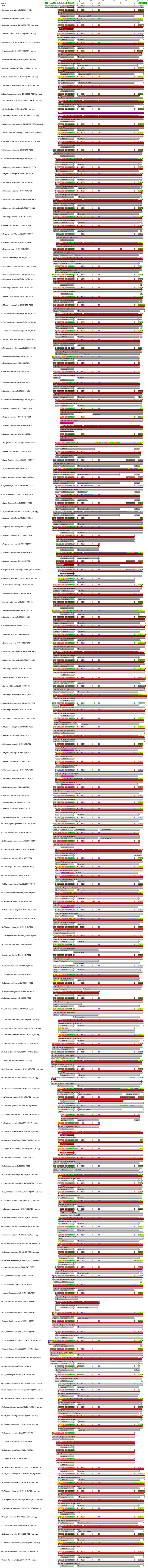

Supplement: Supplementary file 1 — Additional file 1: Annotated sequence alignment of pseudogene repeats found in Solanaceae. Major parts of the trnF gene are marked as D- and T-domains and anticodon in the middle together with bordering 5′ and 3′ acceptor stems. The trnF gene of Nicotiana tabacum is used as a reference sequence to align different pseudogenes. (PDF 4 MB) [file 40064_2013_535_MOESM1_ESM.pdf]
